# Supplementary material for: The order and logic of CD4 versus CD8 lineage choice and differentiation in mouse thymus
Source: Nat Commun. 2021 Jan 4;12:99. doi: 10.1038/s41467-020-20306-w (PMC7782583; doi:10.1038/s41467-020-20306-w)
Supplement: Supplementary file 1 — Supplementary Information [file 41467_2020_20306_MOESM1_ESM.pdf]

The order and logic of CD4 CD8 lineage choice and differentiation in mouse thymus

Mohammad M Karimi[1,5], Ya Guo[1,6], Xiaokai Cui[1], Husayn A Pallikonda[1], Veronika Horková[2], Yi-Fang Wang[1], Sara Ruiz Gil[3], Gustavo Rodriguez-Esteban[3], Irene Robles-Rebollo[1], Ludovica Bruno[1], Radina Georgieva[1], Bhavik Patel[1], James Elliott[1], Marian H Dore[1], Danielle Dauphars[4], Michael S. Krangel[4], Boris Lenhard[1], Holger Heyn[3], Amanda G Fisher[1], Ondřej Štěpánek[2], Matthias Merkenschlager[1*]

[1] MRC London Institute of Medical Sciences, Institute of Clinical Sciences, Faculty of Medicine, Imperial College London, London, UK
[2] Laboratory of Adaptive Immunity, Institute of Molecular Genetics of the Czech Academy of Sciences, Prague, Czech Republic
[3] CNAG-CRG, Centre for Genomic Regulation (CRG), The Barcelona Institute of Science and Technology (BIST), Barcelona, Spain
[4] Department of Immunology, Duke University Medical Center, Durham, NC, USA
[5] Present address: Comprehensive Cancer Centre, School of Cancer & Pharmaceutical Sciences, Faculty of Life Sciences & Medicine, King's College London, London, UK
[6] Present address: School of Life Sciences and Biotechnology, Shanghai Jiao Tong University, Shanghai, China

Address correspondence to matthias.merkenschlager@lms.mrc.ac.uk

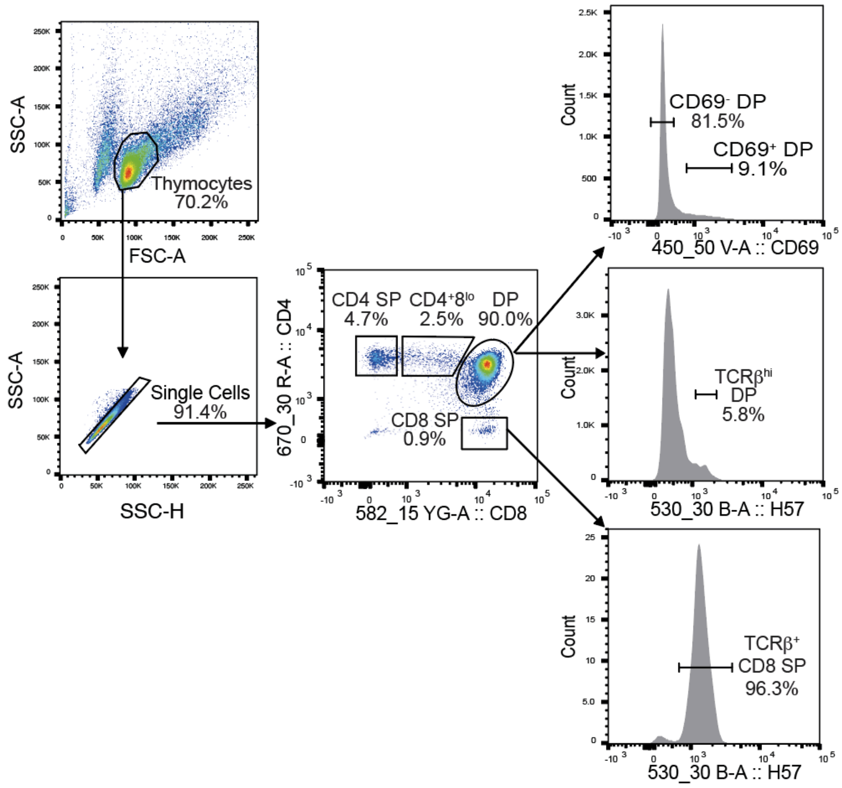

Supplementary Figure 1. Isolation of thymocyte subsets.

Plate layout was designed to represent each sorted population on each plate, enabling analysis of plates as technical replicates. Cells were sorted directly into lysis buffer and SMART-Seq2 libraries were prepared for sequencing of full length transcripts.

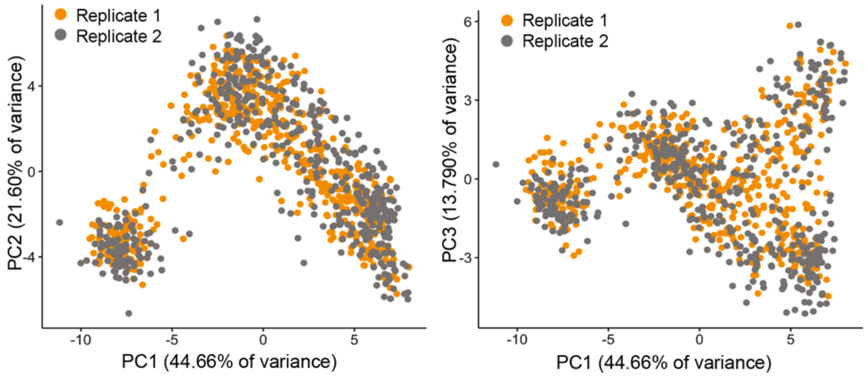

Supplementary Figure 2. PCA analysis of scRNA-seq data - reproducibility between replicates

PCA was performed as in Fig. 2a. Reproducibility between replicates is shown. Source data are provided as a Source Data file.

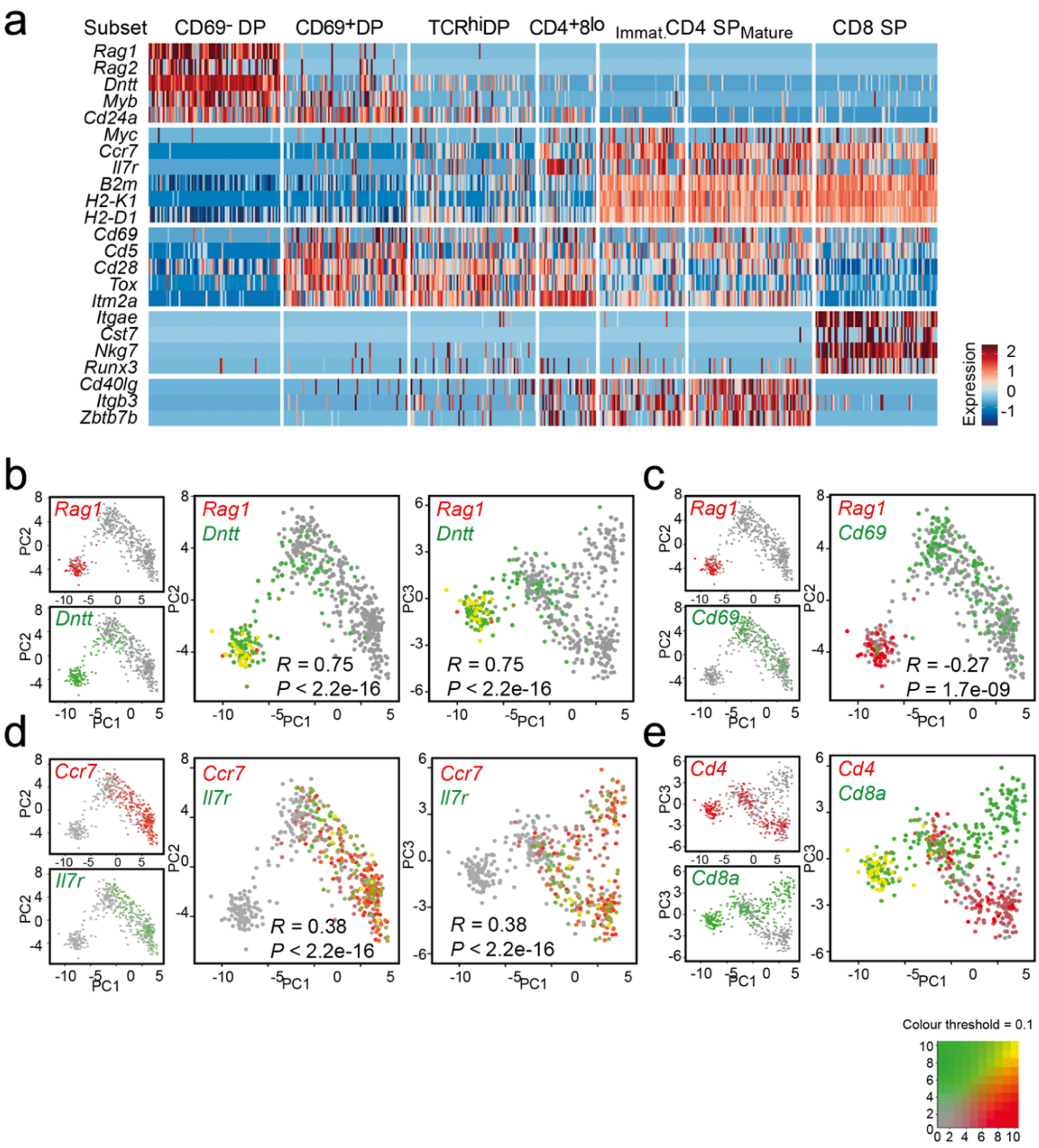

Supplementary Figure 3. Key genes and correlations

a) Heatmap of key genes. See Supplementary Data 2 for an analysis of differential gene expression between sorted thymocyte subsets.

b) *Rag1* (red) and *Dntt* (green) as two-colour dot plots projected onto maps of PC1 versus PC2 and -3. *Rag1* and *Dntt* are expressed in pre-selection thymocytes and silenced by TCR signaling. Source data are provided as a Source Data file.

c) *Rag1* (red) and *Cd69* (green) as two-colour dot plots projected onto maps of PC1 versus PC2. *Rag1* is expressed in pre-selection thymocytes and *Cd69* is induced by TCR signaling Source data are provided as a Source Data file.

d) *Ccr7* (red) and *Il7r* (green) as two-colour dot plots projected onto maps of PC1 versus PC2 and -3. *Ccr7* and *Il7r* expressed are induced during thymocyte maturation. Source data are provided as a Source Data file.

e) *Cd4* (red) and *Cd8a* (green) as two-colour dot plots projected onto maps of PC1 versus PC3. *Cd4* and *Cd8a* are co-expressed in pre-selection thymocytes, undergo expression changes in selection intermediates, and are expressed in a mutually exclusive pattern in CD4 SP and CD8 SP thymocytes. Source data are provided as a Source Data file.

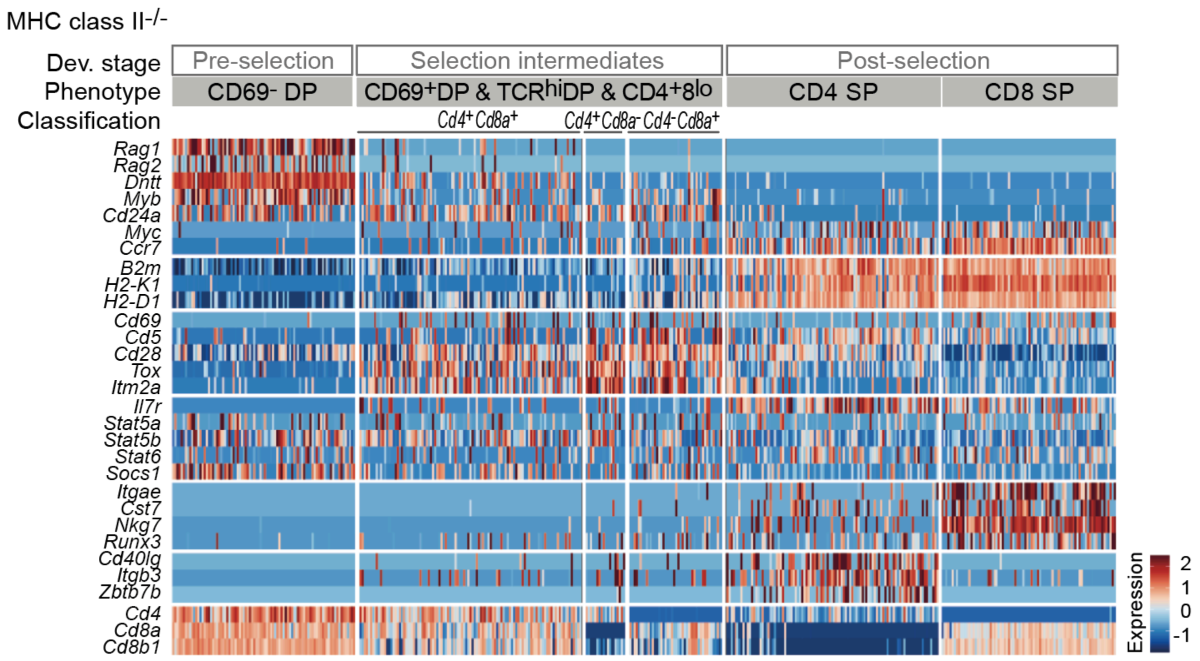

Supplementary Figure 4: Overview of gene expression by individual selection intermediates in MHC class II$^{-/-}$ thymus.

Cells (columns) were classified by *Cd4* and *Cd8a* coreceptor gene expression status, with pre- and post-selection thymocytes as comparators as in Fig. 3c. Cells are grouped by developmental stage (pre-selection, selection intermediates and post-selection) and cell surface phenotype. CD69^{-} DP represent pre-selection thymocytes, pooled CD69^{+} DP, TCRβ^{hi} DP and CD4^{+} CD8^{low} represent selection intermediates, CD4 SP, TCRβ^{hi} CD8 SP represent post-selection thymocytes. Selection intermediates are further classified into *Cd4*$^{+}$*Cd8a*$^{+}$, *Cd4*$^{+}$*Cd8a*$^{-}$ and *Cd4*$^{-}$*Cd8a*$^{+}$ based on scRNA-seq detection of *Cd4* and *Cd8a.*

MHC class II$^{-/-}$

Subset	Surface phenotype	Number
CD69^{-} DP	CD4^{+} CD8^{+} CD69^{-}	88
CD69^{+} DP	CD4^{+} CD8^{+} CD69^{+}	58
TCRβ^{hi} DP	CD4^{+} CD8^{+} TCRβ^{hi}	68
CD4^{+}CD8^{low}	CD4^{+} CD8^{low}	59
CD4 SP	CD4^{+} CD8^{-}	102
CD8 SP	CD4^{-} CD8^{+} TCRβ^{hi}	84

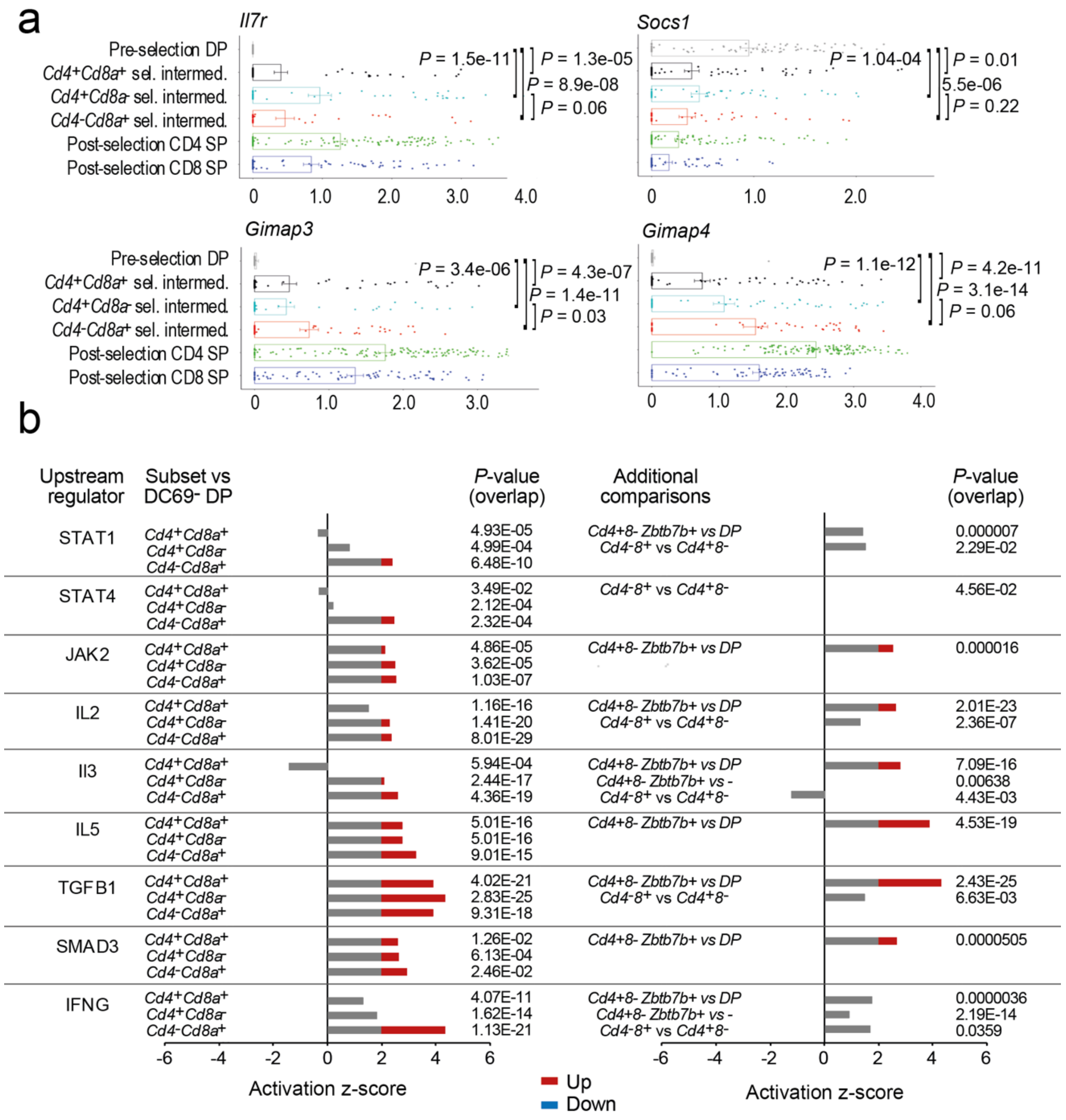

Supplementary Figure 5. Expression of genes related to cytokine signaling by selection intermediates

a) Expression of *Il7r*, *Socs1*, and the targets of IL7R signaling *Gimap3* and *Gmap4* by selection intermediates of the indicated coreceptor status. Means and standard errors are shown. *P*-values are derived by two-sided Wilcoxon rank-sum test. Cell numbers are listed in Supplementary Tables 2 and 3. Source data are provided as a Source Data file.

b) IPA analysis of pathway activity downstream of the indicated cytokines and cytokine signaling molecules. Activation z-scores above 2 and below -2 are considered significant. Selection intermediates versus CD69- DP is shown on the left. Where available, comparisons between subsets of selection intermediates are shown on the right. Cell numbers are listed in Supplementary Tables 2 and 3. Source data are provided as a Source Data file.

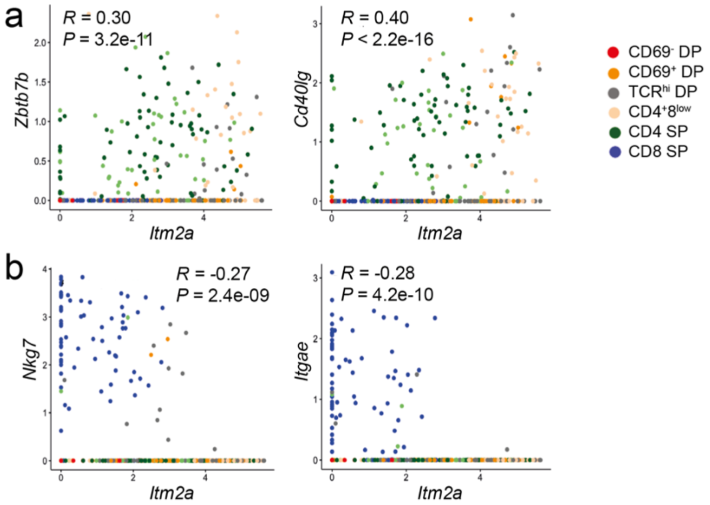

Supplementary Figure 6. Expression of activation versus lineage markers

a) Positive correlation of the activation marker *Itm2a* with the CD4 lineage markers *Zbtb7b* and *Cd40lg* (*R* = 0.30, *P* = 3.2e-11 and *R* = 0.40*, P* < 2.2e-16, respectively). Source data are provided as a Source Data file.

b) Negative correlation of the activation marker *Itm2a* with the CD8 lineage markers *Nkg7* and *Itgae* (*R* = -0.27, *P* = 2.4e-09 and *R* = -0.28*, P* = 4.2e-10, respectively). Source data are provided as a Source Data file.

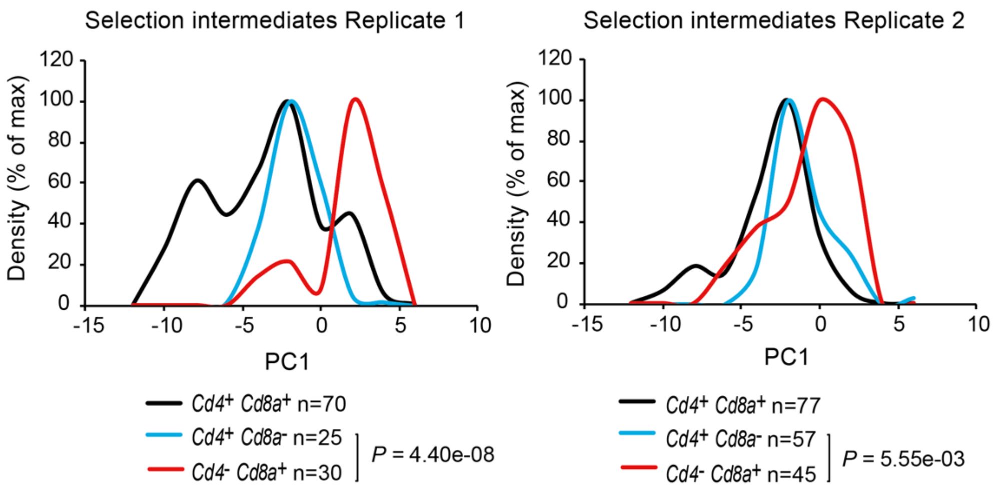

Supplementary Figure 7. The order of coreceptor gene expression by wild-type selection intermediates in independent biological replicates.

The vertical axis shows the number of selection intermediates with the indicated gene expression (*Cd4*$^+$ *Cd8a*$^+$, *Cd4*$^+$ *Cd8a*$^-$, *Cd4*$^-$ *Cd8a*$^+$) normalised to the maximal number of cells detected for each gene expression pattern in separate scRNA-seq replicates. The number of selection intermediates with each coreceptor gene expression pattern is indicated. *P*-values: one-sided Kolmogorov–Smirnov test. Source data are provided as a Source Data file.

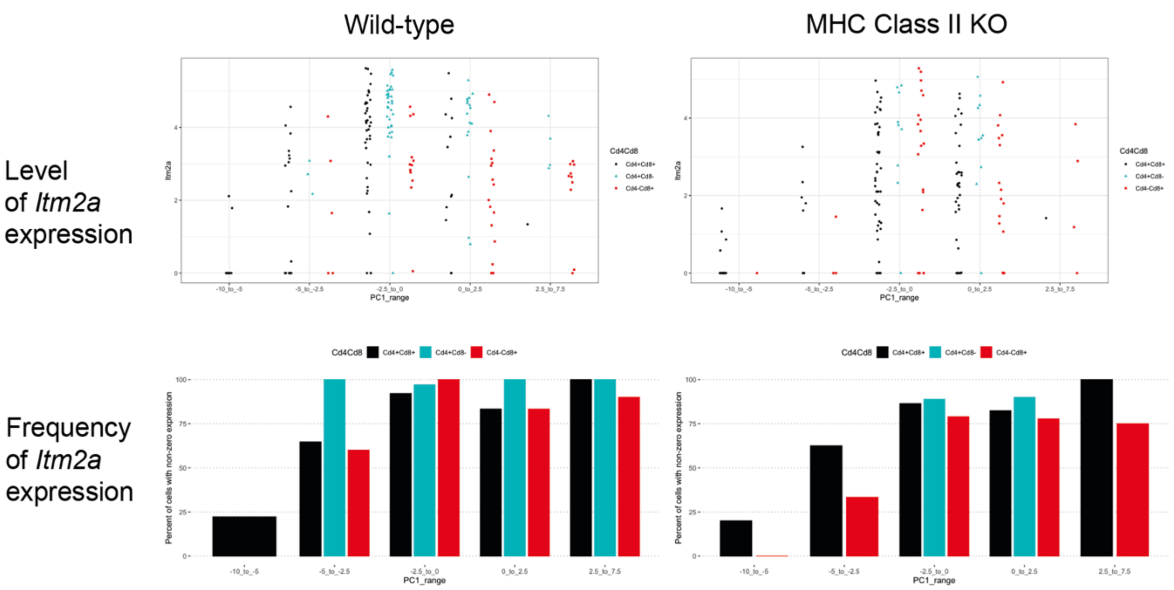

Supplementary Figure 8. Activation marker expression by individual selection intermediates ordered along the maturation trajectory PC1.

Top: The level of mRNA for the activation marker *Itm2a* is shown along the maturation trajectory PC1 for individual selection intermediates classified as *Cd4*$^+$ *Cd8a*$^+$, *Cd4*$^+$ *Cd8a*$^-$, and *Cd4*$^-$ *Cd8a*$^+$ in wild-type (left) and MHC class II-deficient thymus (right).

Bottom: The frequency of cells containing mRNA for the activation marker *Itm2a* detected in subsets of selection intermediates classified as *Cd4*$^+$ *Cd8a*$^+$, *Cd4*$^+$ *Cd8a*$^-$, and *Cd4*$^-$ *Cd8a*$^+$ in wild-type (left) and MHC class II-deficient thymus (right) is shown along the maturation trajectory PC1.

Source data are provided as a Source Data file.

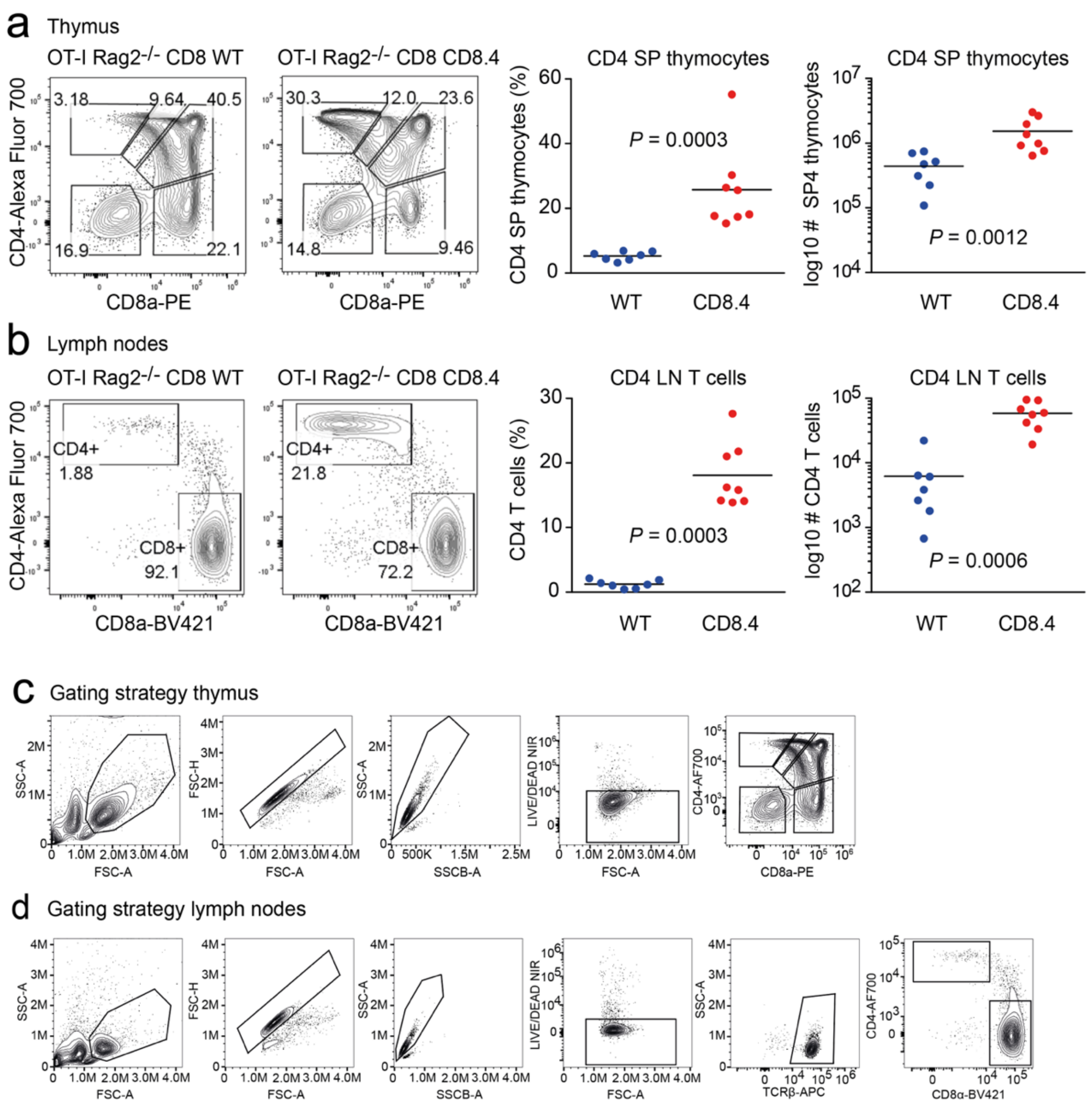

Supplementary Figure 9. Signal strength can undermine CD4/CD8 lineage choice.

a) CD4 CD8 staining representative of OT-I-transgenic *Rag2*$^{-/-}$ thymocytes expressing wild-type Cd8a (left, n=4) or CD8.4 (right, n=5). Percentages and numbers of CD4 SP are shown. *P*-values were calculated by two-sided Mann-Whitney test in GraphPad Prism software. The gating strategy is shown in c). Source data are provided as a Source Data file.

b) CD4 CD8 staining representative of OT-I-transgenic *Rag2*$^{-/-}$ lymph node T cells expressing wild-type Cd8a (left, n=7) or CD8.4 (right, n=8). Percentages and numbers of CD4 T cells relative to total T cells are shown. *P*-values were calculated by two-sided Mann-Whitney test in GraphPad Prism software. The gating strategy is shown in d). Source data are provided as a Source Data file.

c) Gating strategy for thymocytes used in a).

d) Gating strategy for lymph node T cells used in b).

Supplementary Tables

	Aver. transcripts/cell	Aver. reads/cell
Wild type repl. 1	1199.414	677142.7
Wild type repl. 2	2855.474	896571.1
MHC class II-/-	2843.217	839099.7

Supplementary Table 1. Number of transcripts and reads per cell

Subset	Surface phenotype	Rep1	Rep 2	Total
$CD69^-$ DP	$CD4^+$ $CD8^+$ $CD69^-$	92	83	175
$CD69^+$ DP	$CD4^+$ $CD8^+$ $CD69^+$	90	78	168
TCR^{hi} DP	$CD4^+$ $CD8^+$ $TCR\beta^+$	96	79	175
$CD4^+CD8^{low}$	$CD4^+$ $CD8^{low}$	71	36	107
CD4 SP	$CD4^+$ $CD8^-$	113	142	255
CD8 SP	$CD4^-$ $CD8^+$ $TCR\beta^+$	89	77	166

Supplementary Table 2. The numbers of sorted subsets analysed.

	Rep1	Rep 2	Total
CD69- DP	92	83	175
Cd4+ *Cd8a*+ sel. int.	70	77	107
Cd4+ *Cd8a*- sel. int.	125	57	182
Cd4- *Cd8a*+ sel. int.	30	45	75
CD4 SP	113	132	245
CD8 SP	89	77	166

Supplementary Table 3. The numbers of wild-type *Cd4+Cd8a+*, *Cd4+Cd8a* and *Cd4-Cd8a+* selection intermediates analysed.

		CD4SP/CD8SP		
		Up	N/C	Down
$Cd4^+$ $Cd8a^-$	Up	22	44	3
$Cd4^-$ $Cd8a^+$	N/C	326	9234	307
	Down	1	50	14

Supplementary Table 4. Gene expression in CD4 and CD8 SP versus $Cd4^+Cd8a^-$ and $Cd4^-Cd8a^+$ subsets of selection intermediates. Genes up or down in CD4 over CD8 SP (adj P < 0.05 in population RNA-seq) and log2 fold-change > 0.5 between $Cd4^+Cd8a^-$ and $Cd4^-Cd8a^+$ subsets of selection intermediates in scRNA-seq). N/C: No change. P < 2.2e-16, Z = 10.65. Asymptotic Linear-by-Linear Association Test. Data: Var2 (ordered) by Var1 (up < nc < down).
