# Supplementary material for: The order and logic of CD4 versus CD8 lineage choice and differentiation in mouse thymus
Source: Nat Commun. 2021 Jan 4;12:99. doi: 10.1038/s41467-020-20306-w (PMC7782583; doi:10.1038/s41467-020-20306-w)
Supplement: Supplementary file 3 — Reporting Summary [file 41467_2020_20306_MOESM3_ESM.pdf]

nature research

Corresponding author(s): M Merkenschlager

Last updated by author(s): Oct 29, 2020

Reporting Summary

Nature Research wishes to improve the reproducibility of the work that we publish. This form provides structure for consistency and transparency in reporting. For further information on Nature Research policies, see Authors & Referees and the Editorial Policy Checklist .

Statistics

For all statistical analyses, confirm that the following items are present in the figure legend, table legend, main text, or Methods section.

n/a	Confirmed	
☐	☒	The exact sample size (*n*) for each experimental group/condition, given as a discrete number and unit of measurement
☐	☒	A statement on whether measurements were taken from distinct samples or whether the same sample was measured repeatedly
☐	☒	The statistical test(s) used AND whether they are one- or two-sided<br>*Only common tests should be described solely by name; describe more complex techniques in the Methods section.*
☒	☐	A description of all covariates tested
☐	☒	A description of any assumptions or corrections, such as tests of normality and adjustment for multiple comparisons
☐	☒	A full description of the statistical parameters including central tendency (e.g. means) or other basic estimates (e.g. regression coefficient) AND variation (e.g. standard deviation) or associated estimates of uncertainty (e.g. confidence intervals)
☐	☒	For null hypothesis testing, the test statistic (e.g. *F*, *t*, *r*) with confidence intervals, effect sizes, degrees of freedom and *P* value noted<br>*Give P values as exact values whenever suitable.*
☒	☐	For Bayesian analysis, information on the choice of priors and Markov chain Monte Carlo settings
☒	☐	For hierarchical and complex designs, identification of the appropriate level for tests and full reporting of outcomes
☒	☐	Estimates of effect sizes (e.g. Cohen's *d*, Pearson's *r*), indicating how they were calculated

Our web collection on statistics for biologists contains articles on many of the points above.

Software and code

Policy information about availability of computer code

Data collection	DIVA flow cytometry software version 8.0.2 (BD Biosciences)
Data analysis	• scRNA-seq reads were aligned to the mm10 mouse genome with TopHat2 version 2.1.1<br>• read counts for genes were calculated using velocyto version 0.17<br>• Seurat v3 pre-processing (log-normalization using VST method) was applied on the count matrix<br>• Seurat v3 standard integration workflow was performed for integrated analysis of wild-type and MHC class II-/- data sets<br>• Batch-corrected expression matrix, PCA, heat maps, and differentially expressed genes were all generated by Seurat v3<br>• Slingshot was used for trajectory inference and pseudotime analysis<br>• Custom Rmd scripts used for generating figures are available from Github: https://github.com/LMSBioinformatics/ScRNAseq-SMARTer-Analysis<br>• FlowJo v10 (TreeStar Inc)<br>• GraphPad Prism v5.04 (GraphPad Software)

For manuscripts utilizing custom algorithms or software that are central to the research but not yet described in published literature, software must be made available to editors/reviewers. We strongly encourage code deposition in a community repository (e.g. GitHub). See the Nature Research guidelines for submitting code & software for further information.

Data

Policy information about availability of data

All manuscripts must include a data availability statement. This statement should provide the following information, where applicable:

- Accession codes, unique identifiers, or web links for publicly available datasets
- A list of figures that have associated raw data
- A description of any restrictions on data availability

scRNA-seq data has been deposited at GEO under accession number GSE149207 https://www.ncbi.nlm.nih.gov/geo/query/acc.cgi?acc=GSE149207

Population RNA-seq data has been deposited at GEO under accession number GSE154670 https://www.ncbi.nlm.nih.gov/geo/query/acc.cgi?acc=GSE154670 . All other data are included in the supplemental information or available from the authors upon reasonable requests.

https://www.ncbi.nlm.nih.gov/geo/query/acc.cgi?acc=GSE154670

Field-specific reporting

Please select the one below that is the best fit for your research. If you are not sure, read the appropriate sections before making your selection.

- [x] Life sciences
- [ ] Behavioural & social sciences
- [ ] Ecological, evolutionary & environmental sciences

For a reference copy of the document with all sections, see nature.com/documents/nr-reporting-summary-flat.pdf

Life sciences study design

All studies must disclose on these points even when the disclosure is negative.

Sample size — Sample sizes were determined based on expected effect sizes, technical limitations and budget considerations. The significance of the results obtained suggests that the chosen sample sizes are appropriate

Data exclusions — Data were excluded only for the SMARTer sequencing replicates, and only if the total read counts were < 500000 or > 1500000

Replication — scRNA-seq: Wild-type thymus n=2, MHC class II ko n = 1. Bulk RNA-seq n = 3 per sorted subset.
Replicates Flow cytometric analysis of OT-I TCR transgenic mice (Supplementary Figure 8): Thymus wild-type CD8 n=4, CD8.4 n=5. Lymph node T cells wild-type Cd8 n=7, CD8.4 n=8)

Randomization — Randomization was not performed because groups were defined by genotype.

Blinding — No formal blinding was considered necessary as the data analysts lacked prior knowledge of the underlying biological process that could have biased their expectations

Reporting for specific materials, systems and methods

We require information from authors about some types of materials, experimental systems and methods used in many studies. Here, indicate whether each material, system or method listed is relevant to your study. If you are not sure if a list item applies to your research, read the appropriate section before selecting a response.

Materials & experimental systems

n/a	Involved in the study	
[ ]	[x]	Antibodies
[x]	[ ]	Eukaryotic cell lines
[x]	[ ]	Palaeontology
[ ]	[x]	Animals and other organisms
[x]	[ ]	Human research participants
[x]	[ ]	Clinical data

Methods

n/a	Involved in the study	
[x]	[ ]	ChIP-seq
[ ]	[x]	Flow cytometry
[x]	[ ]	MRI-based neuroimaging

Antibodies

Antibodies used — CD4-APC, TCRb-FITC, CD69-BV421 (Pharmingen) or CD4-Alexa Fluor 700, CD8a-PE, CD24-FITC and LIVE/DEAD NIR (ThermoFisher)

Validation — The antibodies were manufacturer-validated on mouse T cells.

Animals and other organisms

Policy information about studies involving animals; ARRIVE guidelines recommended for reporting animal research

Laboratory animals — C57BL/6 and MHC class II-deficient male or female mice were maintained under SPF conditions with chow and water ad libitum, 21 degrees Celsius and 12h light/dark cycle and were killed by cervical dislocation at 6 weeks of age for use as tissue donors

Wild animals — No wild animals were used in this study

Field-collected samples | No field-collected samples were used in this study

Ethics oversight | The maintenance of mice used as source for tissue in this study was approved by the Home Office, UK, and the Imperial College Animal Welfare and Ethical Review Body (AWERB)

Note that full information on the approval of the study protocol must also be provided in the manuscript.

Flow Cytometry

Plots

Confirm that:

- [x] The axis labels state the marker and fluorochrome used (e.g. CD4-FITC).
- [x] The axis scales are clearly visible. Include numbers along axes only for bottom left plot of group (a 'group' is an analysis of identical markers).
- [x] All plots are contour plots with outliers or pseudocolor plots.
- [x] A numerical value for number of cells or percentage (with statistics) is provided.

Methodology

Sample preparation	Thymocyte cell suspensions were stained for 20 minutes at room temperature with directly conjugated antibodies to CD4-APC, CD8a-PE, TCRb-BV421 and CD69-FITC (BD-Pharmingen).
Instrument	Single cells were sorted into 96 well plates containing lysis buffer using a FACSAria Fusion flow cytometer (BD Biosciences) and the gates depicted in Supplementary Fig. 1
Software	Flow cytometry standard files were analyzed with FlowJo v10 (TreeStar Inc) analysis software.
Cell population abundance	The abundance of sorted populations is depicted in Supplementary Fig. 1. Sort purity for populations was >98%. Sort purity for single cells was not checked. Single cell deposition was checked by sorting limited amount of substrate into enzyme-containing wells followed by colorimetric readout
Gating strategy	The gating strategy was: live gate, doublet exclusion, fluorescence gates as depicted in Supplementary Fig. 1

- [x] Tick this box to confirm that a figure exemplifying the gating strategy is provided in the Supplementary Information.
