# Supplementary material for: The order and logic of CD4 versus CD8 lineage choice and differentiation in mouse thymus
Source: Nat Commun. 2021 Jan 4;12:99. doi: 10.1038/s41467-020-20306-w (PMC7782583; doi:10.1038/s41467-020-20306-w)
Supplement: Supplementary file 4 — Description of Additional Supplementary Files [file 41467_2020_20306_MOESM4_ESM.pdf]

Description of Additional Supplementary Files

Supplementary Data 1. Highly variable genes used for PCA analysis after removal of *Cd4, Cd8a*, and *Cd8b1* (excel file).

Supplementary Data 2. Differential gene expression of sorted thymocyte subsets (excel file).

Supplementary Data 3. Genes that are transiently up-or downregulated in selection intermediates (activation) or differentially expressed between CD4 and CD8 SP (lineage identity) identified by bulk RNA-seq (excel file).

Supplementary Data 4. Differential gene expression between selection intermediates classified by coreceptor status (excel file).
